# Correction: Karpova et al. A Feature of the Crystalline and Amorphous Structure of Ultra Thin Fibers Based on Poly(3-hydroxybutyrate) (PHB) Containing Minor Concentrations of Hemin and a Complex of Tetraphenylporphyrin with Iron. *Polymers* 2022, *14*, 4055

**DOI:** 10.3390/polym15092029

**Published:** 2023-04-25

**Authors:** Svetlana G. Karpova, Ivetta A. Varyan, Anatoly A. Olkhov, Polina M. Tyubaeva, Anatoly A. Popov

**Affiliations:** 1 Department of Biological and Chemical Physics of Polymers, Emanuel Institute of Biochemical Physics, Russian Academy of Sciences, 4 Kosygina Street, 119334 Moscow, Russia; ivetta.varyan@yandex.ru (I.A.V.); aolkhov72@yandex.ru (A.A.O.); polina-tyubaeva@yandex.ru (P.M.T.); anatoly.popov@mail.ru (A.A.P.); 2 Academic Department of Innovational Materials and Technologies Chemistry, Plekhanov Russian University of Economics, 36 Stremyanny Lane, 117997 Moscow, Russia

**Addition of an Author**

**Polina M. Tyubaeva** was not included as an author in the original publication [1]. The corrected Author Contributions Statement appears here. The authors apologize for any inconvenience caused and state that the scientific conclusions are unaffected. This correction was approved by the Academic Editor. The original publication has also been updated.

**Author Contributions**

Conceptualization, S.G.K. and I.A.V.; investigation, S.G.K., P.M.T. and I.A.V.; resources, I.A.V. and P.M.T.; data curation, A.A.O.; writing—original draft preparation, S.G.K. and A.A.P.; writing—review and editing, A.A.P. and I.A.V.; visualization, S.G.K.; supervision, S.G.K.; project administration, I.A.V.; funding acquisition, I.A.V. All authors have read and agreed to the published version of the manuscript.
